# The Enhanced Performance of NiCuOOH/NiCu(OH)_2_ Electrode Using Pre-Conversion Treatment for the Electrochemical Oxidation of Ammonia

**DOI:** 10.3390/molecules29102339

**Published:** 2024-05-16

**Authors:** Xuejiao Yin, Jiaxin Wen, Jujiao Zhao, Ran An, Ruolan Zhang, Yin Xiong, Yanzong Tao, Lingxin Wang, Yuhang Liu, Huanyu Zhou, Yuanyuan Huang

**Affiliations:** 1 School of Architecture and Engineering, Chongqing Industry Polytechnic College, Chongqing 401120, China; 2 College of Environment and Resources, Chongqing Technology and Business University, Chongqing 400067, China; zhaojujiao@ctbu.edu.cn (J.Z.);; 3 Chongqing Baihan Wastewater Treatment Co., Ltd., Chongqing 400000, China; 13594739722@163.com (Y.X.);; 4 School of Civil Engineering and Architecture, Chongqing University of Science and Technology, Chongqing 401331, China; 5 Green Intelligence Environmental School, Yangtze Normal University, Chongqing 408100, China; 6 Key Laboratory of Hydraulic and Waterway Engineering of the Ministry of Education, School of River and Ocean Engineering, Chongqing Jiaotong University, Chongqing 400074, China; 7 Chongqing Academy of Science and Technology, Chongqing 401120, China

**Keywords:** electrochemical oxidation of ammonia, nickel–copper oxyhydroxide, wastewater treatment, hydrogen production

## Abstract

Electrochemical oxidation of ammonia is an attractive process for wastewater treatment, hydrogen production, and ammonia fuel cells. However, the sluggish kinetics of the anode reaction has limited its applications, leading to a high demand for novel electrocatalysts. Herein, the electrode with the in situ growth of NiCu(OH)_2_ was partially transformed into the NiCuOOH phase by a pre-treatment using highly oxidative solutions. As revealed by SEM, XPS, and electrochemical analysis, such a strategy maintained the 3D structure, while inducing more active sites before the in situ generation of oxyhydroxide sites during the electrochemical reaction. The optimized NiCuOOH-1 sample exhibited the current density of 6.06 mA cm^−2^ at 0.5 V, which is 1.67 times higher than that of NiCu(OH)_2_ (3.63 mA cm^−2^). Moreover, the sample with a higher crystalline degree of the NiCuOOH phase exhibited lower performance, demonstrating the importance of a moderate treatment condition. In addition, the NiCuOOH-1 sample presented low selectivity (<20%) towards NO_2_^−^ and stable activity during the long-term operation. The findings of this study would provide valuable insights into the development of transition metal electrocatalysts for ammonia oxidation.

## 1. Introduction

Ammonia, a compound synthesized in vast quantities for fertilizers, industrial processes, and various agricultural activities, has inadvertently become a severe environmental hazard [1,2,3]. Excess ammonia in wastewater can precipitate a host of environmental problems, notably eutrophication. This phenomenon, characterized by an overgrowth of algae due to high nutrient concentrations, disrupts aquatic ecosystems and significantly deteriorates water quality [4]. Moreover, a high concentration of ammonia can act as an irritant and cause severe damage to the respiratory tract, eyes, and skin [5]. Thus, tackling the issue of ammonia pollution is an environmental imperative that demands immediate and innovative solutions.

Against this backdrop, the electrochemical oxidation of ammonia has emerged as a promising avenue for its degradation [3,6,7]. More importantly, this process has the added advantage of simultaneously generating hydrogen, a clean and renewable energy carrier [2]. This dual benefit renders the electrochemical oxidation of ammonia to be an appealing strategy in the broader context of sustainable energy and environmental conservation. The overall reaction is defined as follows [8,9]:

NH_3_(aq) + 3OH^−^ → 1/2N_2_ + 3H_2_O + 3e^−^ E^0^ = −0.77 V vs. SHE (1)

3H_2_O + 3e^−^ → 3/2H_2_ + 3OH^−^ E^0^ = −0.83 V vs. SHE (2)

NH_3_(aq) → 1/2N_2_ + 3/2H_2_ E^0^_cell_ = 0.06 V (3)

One of the attractive aspects of this reaction is that it can occur at a surprisingly low potential of just 0.06 V, an attribute that makes this process energy-efficient and potentially economically feasible [10]. Nevertheless, the efficiency and practical applicability of the electrochemical oxidation of ammonia largely hinge upon the choice and design of the catalyst involved [10]. Traditionally, catalysts for this process have relied heavily on noble metals [11]. The overpotential of Pt for the electrochemical oxidation of ammonia is approximately 0.48 V; thus, Pt has been regarded as the most effective ammonia oxidation electrocatalyst for an extended period [12]. Nevertheless, Pt undergoes fast deactivation during the electrochemical oxidation of ammonia since the binding energy of N atoms on the Pt surface is too strong, impeding its practical applications. To overcome this demerit, some Pt-based complexes such as PtRh/C [13] and PtSnO_2_/C [8] have been developed. Although the stability of these catalysts has been improved, the high cost and scarcity of noble metals still seriously impede their large-scale applications, motivating the quest for cost-effective alternatives [14,15,16].

Research on non-noble metals and their compounds has been pursued as an avenue to circumvent these challenges. Nickel-based catalysts have shown some promise due to their abundance, low cost, and reasonable activity [17,18,19,20,21]. Recently, it is widely reported that the NiCu-based bi-metal catalysts show better activity than other bi-metal catalysts and single-metal catalysts, suggesting its potential for applications [6,22,23,24,25,26,27,28,29]. However, the performance of these materials still needs to be improved.

It is presumed by several studies that the actual active phase of the NiCu-based catalysts is the NiCu oxyhydroxide phase (NiCuOOH), which is formed in situ during the electrochemical reaction [17,21,27,30]. The formation of NiCuOOH under anodic conditions has been regarded as the key step before the electrochemical oxidation of ammonia. This improvement is largely attributed to the unique electronic structure and greater availability of active sites in the NiCuOOH phase [22,31].

Yet, the inherent challenge with such catalysts lies in the fact that the NiCu atoms on the surface of electrode might not be totally converted into the NiCuOOH phase in the electrochemical oxidation process. Compared to the pure NiCuOOH phase, the amount of in situ-generated NiCuOOH active sites might be limited, potentially constraining the performance of these catalysts. Furthermore, the in situ-formed NiCuOOH generally exhibited low crystallinity. Despite some studies reporting that the non-crystalline materials exhibit better electrochemical activity [32], very few studies have assessed the influence of the crystalline degree for the electrochemical oxidation of ammonia.

According to the current insights in this field, it seems that a pure NiCuOOH electrode should be a better choice than other NiCu-based electrocatalysts. However, the NiCuOOH electrode did not exhibit superior performance than other electrodes [22,27]. One of the main reasons is that the morphology of NiCuOOH is difficult to adjust. It is well known that the morphology of catalysts plays an important role in the electrochemical reaction [33]. The 3D nanostructure of catalysts could contribute to their effectiveness by ensuring a high surface area and shorter diffusion paths. These nanostructures enhance reactant access to active sites and product removal, thereby accelerating the overall reaction rate [34].

Thus, based on all above facts, the NiCu(OH)_2_ electrode with a well-defined 3D structure was synthesized, and it was treated by a pre-treatment before the electrochemical reaction to form the 3D NiCuOOH phase with different crystalline degrees. The pre-treatment process partially transforms the NiCu(OH)_2_ into the active phase NiCuOOH while preserving the beneficial 3D nanostructure. This approach has led to a significant improvement in catalyst performance, being a major step forward in the practical application of these materials for the electrochemical oxidation of ammonia.

## 2. Results and Discussion

### 2.1. Characterization of Catalysts

**Figure 1:**
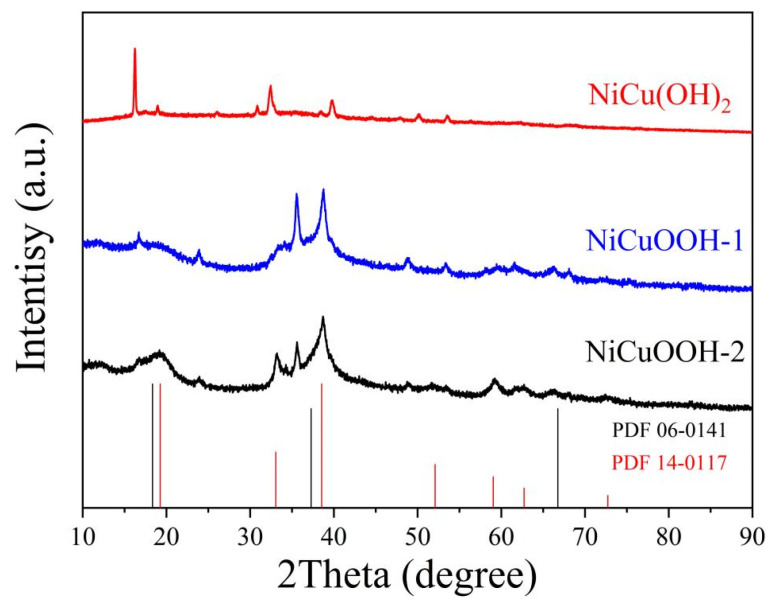
The XRD patterns of NiCu(OH)_2_, NiCuOOH-1, and NiCuOOH-2.

The crystalline structures of the prepared samples were investigated using XRD, and the obtained patterns are presented in Figure 1. For the as-prepared NiCu(OH)_2_ sample, the observed XRD pattern shows characteristic peaks located at 16.7°, 32.4°, and 39.8°, which correspond to the reported NiCu(OH)_2_, indicating its successful synthesis [24]. In the pattern of the NiCuOOH-1 sample, the intensity of the peak located at around 16.7° corresponding to the (020) facet of Cu(OH)_2_ (JCPDS no. 13-0420) significantly decreased. All the other peaks belonging to NiCu(OH)_2_ also present lower intensity. Meanwhile, the peaks corresponding to the (002) facet of NiCuOOH could be observed [35,36], demonstrating the partial transformation of NiCu(OH)_2_ into NiCuOOH as a result of our pre-treatment process. The XRD pattern of the NiCuOOH-2 sample, which is prepared by a higher concentration of KOH and K_2_S_2_O_8_, was also collected, and it shows peaks exclusively matching the NiCuOOH phase. These results illustrate the impact of the pre-treatment process on the phase transformation of NiCu(OH)_2_ into NiCuOOH and indicate that the NiCuOOH phase was successfully formed after the pre-treatment process.

**Figure 2:**
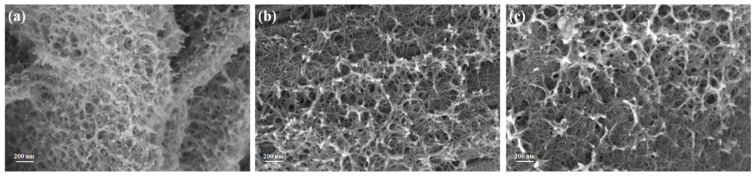
(**a**) The SEM image of NiCu(OH)_2_, (**b**) the SEM image of NiCuOOH-1, and (**c**) the SEM image of NiCuOOH-2.

The morphology of the samples was observed by SEM (Figure 2). The SEM image (Figure 2a) of NiCu(OH)_2_ clearly reveals the porous nanostructure consisting of overlapped nanorods, which is similar to that of reported studies [24]. Interestingly, the SEM images of the pre-treated samples, NiCuOOH-1 and NiCuOOH-2, also exhibit a similar nanostructure. Despite the phase transformation from NiCu(OH)_2_ to NiCuOOH that occurs during the pre-treatment process, the overall morphology of the samples appears to be largely unchanged.

**Figure 3:**
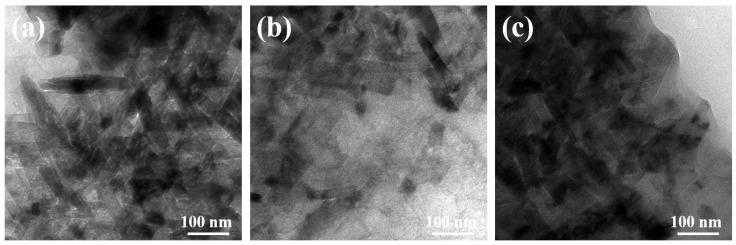
(**a**) The TEM image of NiCu(OH)_2_, (**b**) the TEM image of NiCuOOH-1, and (**c**) the TEM image of NiCuOOH-2.

To further investigate the microstructure of the samples, TEM was conducted, and the images are depicted in Figure 3. The powder of each sample was stripped from the carbon cloth by the ultrasonic treatment used for the TEM measurement. It could be noticed that NiCuOOH-1 and NiCuOOH-2 also present a similar nanostructure, further indicating that the pre-treatment process did not change the morphology of these samples. The length of these nanorods is approximately 100 to 300 nm; meanwhile, their width is approximately 20 to 50 nm. Based on the XRD, SEM, and TEM results, it is suggested that the pre-treatment method successfully induces the phase transformation while preserving the beneficial nanostructure.

**Figure 4:**
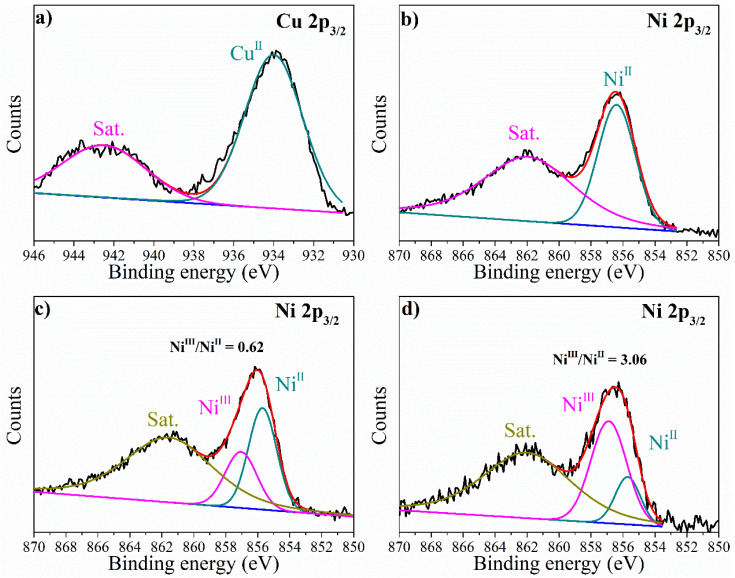
The high-resolution XPS spectra of (**a**) Cu 2p_3/2_ of NiCuOOH-1, (**b**) Ni 2p_3/2_ of NiCu(OH)_2_, (**c**) Ni 2p_3/2_ of NiCuOOH-1, and (**d**) Ni 2p_3/2_ of NiCuOOH-2.

The surface composition and chemical states of the prepared samples were investigated using XPS. The XPS survey spectra (Figure S1) confirmed the presence of Ni, Cu, and O in all three samples, without any noticeable impurities. The high-resolution spectra of Cu 2p_3/2_ with the peak located at 934.5 ± 0.3 eV could be ascribed to the presence of Cu(II) [23,37]. All the three samples showed similar spectra, as shown in Figure 4a and Figure S2. For the high-resolution Ni spectrum of the NiCu(OH)_2_ sample (Figure 4b), peaks at 856.4 and 862.0 eV corresponding to Ni 2p_3/2_ and its satellite peak of β-Ni(OH)_2_ could be observed [18,23], which is in accordance with the XRD results. In the NiCuOOH-1 sample, the Ni 2p_3/2_ showed the presence of both Ni^II^ and Ni^III^, indicating the partial transformation of NiCu(OH)_2_ to the NiCuOOH phase during the pre-treatment [18,38,39]. NiCuOOH-2, the sample treated with a solution having stronger oxidizing properties, presented the Ni^III^/Ni^II^ value of 3.06; meanwhile, the Ni^III^/Ni^II^ value of NiCuOOH-1 is just 0.62, indicating the increased amount of Ni^III^ on the surface of NiCuOOH-2.

Based on the XRD and XPS results, it could be concluded that the pre-treatment process reported in this study could successfully transform NiCu(OH)_2_ into the NiCuOOH phase, and the crystalline degree can be adjusted.

### 2.2. Electrochemical Performance of Samples

The NiCu(OH)_2_ with different Ni:Cu ratios was evaluated by LSV, and the 8:2 sample presented the best performance (Figure S3), which is in agreement with the results reported by other studies [22,24,26]. The electrochemical activity of NiCu(OH)_2_, NiCuOOH-1, and NiCuOOH-2 towards the oxidation of ammonia was first investigated using LSV, as shown in Figure S4a. In the electrolyte containing 0.1 mol/L KOH and 0.1 mol/L NH_3_, all the three samples presented a larger current density than those in the 0.1 mol/L KOH, indicating their activity towards the electrochemical oxidation of ammonia. The onset potentials of the three samples are all around 0.38–0.4 V (the potential with the current density of 1 mA cm^−2^ was treated as the onset potential). Both NiCuOOH-1 and NiCuOOH-2 present the oxidation peak after the onset potential in the solution without 0.1 mol/L NH_3_, and the current density decreases with the increase in potential in this pure KOH solution until the potential is higher than 0.53 V. These peaks could be attributed to the oxidation of Ni and/or Cu on the electrode, which is also reported by other studies [22]. Since the amount of Ni and Cu on the electrode is limited, the current density decreased even with a higher potential in the potential range without other chemical reactions. The increase in current density after 0.53 V could be due to water electrolysis. With the presence of NH_3_, the current densities of NiCuOOH-1 and NiCuOOH-2 increase in a linear manner when the potential is higher than 0.38 V. These results indicate that the oxidation of ammonia in this potential range. It can be noticed that NiCuOOH-1 presented better activity than NiCuOOH-2 for ammonia electrolysis; meanwhile, their performance for water electrolysis is quite similar. They exhibited significantly larger current density than NiCu(OH)_2_ for both water electrolysis and ammonia electrolysis. For investigating the kinetics of these samples for the electrochemical oxidation of ammonia, Tafel slopes were calculated, as shown in Figure S4b. The Tafel slope values of NiCu(OH)_2_, NiCuOOH-1, and NiCuOOH-2 are 151, 76, and 110 mV decade^−1^, respectively. The NiCuOOH-1 presents the smallest Tafel slope value (76 mV decade^−1^), indicating its faster reaction kinetics compared to those of NiCu(OH)_2_ and NiCuOOH-2.

**Figure 5:**
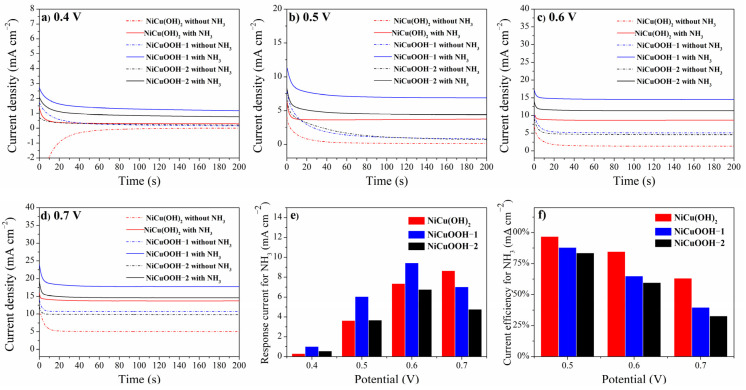
(**a**–**d**) Chronoamperogram of three samples at 0.4 V, 0.5 V, 0.6 V, and 0.7 V, respectively, in 0.1 mol/L KOH with or without 0.1 mol/L NH_3_ and (**e**,**f**) the calculated response current towards ammonia and the current efficiency for ammonia oxidation.

To further reveal the performance of three samples for the electrochemical oxidation of ammonia, chronoamperogram at different potentials was obtained (Figure 5). By maintaining the working voltage for a relatively long time, the current obtained in the chronoamperogram tests is close to the actual performance of the samples. The current density values obtained in 0.1 mol/L KOH with 0.1 mol/L NH_3_ minus those obtained in only 0.1 mol/L KOH were regarded as the response current density towards electrochemical oxidation of ammonia, and the current efficiency was calculated by dividing the response current density by the current density obtained in 0.1 mol/L KOH with 0.1 mol/L NH_3_. For all the samples, the response current density increased when the working potential increased from 0.4 V to 0.6 V. At 0.4 V, the response current densities of NiCu(OH)_2_, NiCuOOH-1, and NiCuOOH-2 are 0.30, 1.02, and 0.56 mA cm^−2^, respectively, indicating the better performance of NiCuOOH-1 under this voltage. Notably, NiCuOOH-1 exhibited the response current density of 6.06 mA cm^−2^ towards ammonia oxidation at 0.5 V, while its value for NiCu(OH)_2_ is just 3.63 mA cm^−2^. Although the current efficiency of NiCuOOH-1 for ammonia oxidation is 87.9%, which is slightly lower than that of NiCu(OH)_2_ (96.8%), a much higher response current density indicates that NiCuOOH-1 shows better performance than NiCu(OH)_2_. However, NiCuOOH-2, which exhibited a higher crystalline degree, presented a response current density of just 3.68 mA cm^−2^ and current efficiency of just 83.6% at 0.5 V, both of which are lower than those of NiCuOOH-1. By further increasing the crystalline degree of samples resulted in worse activity, demonstrating that a moderate treatment condition is necessary for improving the performance.

Further increasing the working potentials to 0.6 V, a response current density of 9.45 mA cm^−2^ could be obtained on NiCuOOH-1, which is also higher than those of NiCu(OH)_2_ (7.37 mA cm^−2^) and NiCuOOH-1 (6.78 mA cm^−2^). However, current efficiency significantly decreased to 65.0%. At 0.7 V, the current efficiency of NiCuOOH-1 further decreased to 40.0%; meanwhile, the response current density also decreased to 7.02 mA cm^−2^. This could be attributed to the enhanced oxygen evolution reaction at high working potentials, in accordance with the reported studies [29,30]. Thus, based on the results shown above, 0.5 V was suggested to be used as the optimized working potential in the following experiments.

**Figure 6:**
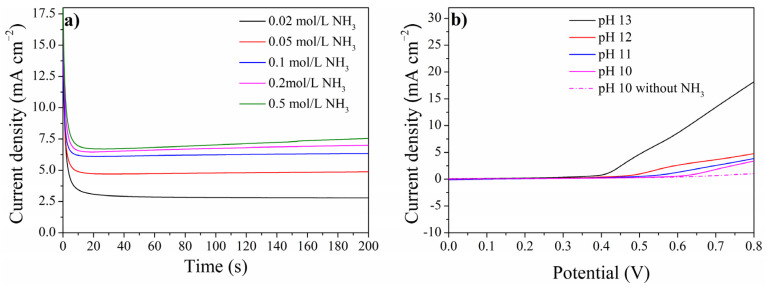
(**a**) Chronoamperogram of NiCuOOH-1 with different ammonia concentrations at 0.5 V and (**b**) the LSV curves of NiCuOOH-1 at different pHs.

To further evaluate the performance of NiCuOOH-1, chronoamperogram were conducted at 0.5 V with different ammonia concentrations. As shown in Figure 6a, the current density increased by increasing the ammonia concentration, further indicating that the increased current originates from the electrochemical oxidation of ammonia. By increasing the ammonia concentration from 0.02 mol/L to 0.05 mol/L, the current density increased from 2.79 mA cm^−2^ to 4.86 mA cm^−2^. However, it could be noticed that the current density for 0.5 mol/L ammonia is just slightly higher than that for 0.2 mol/L ammonia, which could be attributed to the limited ammonia diffusion in the solution [17].

The performance of NiCuOOH-1 at different pH values was also tested, as shown in Figure 6b. Even at pH 10, the NiCuOOH-1 exhibited an obvious response current for ammonia oxidation. Despite NiCuOOH-1 showing activity in the pH range of 10 to 14, it could be noticed that the pH significantly affects the performance of NiCuOOH-1. The observed current density at 0.7 V decreased from 13.42 mA cm^−2^ (pH 13) to 3.64 mA cm^−2^ (pH 12). As shown in Equation (1), the oxidation of ammonia on anode needs the participation of OH^−^. The higher activity at a higher pH indicates the key role of OH^−^ in the electrochemical oxidation of ammonia, as reported by other studies [7,40].

### 2.3. Ammonia Electrolysis at Different Voltages

**Figure 7:**
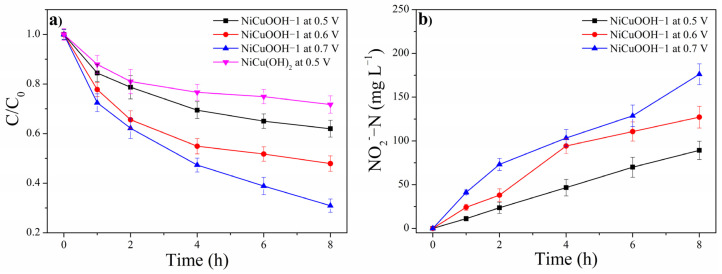
(**a**) NH_3_ removal in a two-chamber reactor by NiCuOOH-1 and NiCu(OH)_2_ at different voltages with an initial NH_3_ concentration of 0.1 mol/L and (**b**) the generation of NO_2_^−^ by NiCuOOH-1 at different voltages.

The selectivity of electrochemical oxidation of ammonia is very important since NO_2_^−^ and NO_3_^−^ are still regarded as pollutants that could be harmful to human health and the environment. The removal of NH_3_ with NiCuOOH-1 as the working electrode in a two-chamber reactor was conducted at 0.5 V, 0.6 V, and 0.7 V, respectively. The ammonia removal with a NiCu(OH)_2_ electrode was also evaluated at 0.5 V as the counter experiment to reveal the performance of NiCuOOH-1. The initial concentration of NH_3_ is 0.1 mol/L (1400 mg/L N). As depicted in Figure 7a, within 8 h, 38.02% of NH_3_ could be removed at 0.5 V by NiCuOOH-1, while 28.26% can be removed by NiCu(OH)_2_, further indicating the better performance of NiCuOOH-1. The NH_3_ removal rate of NiCuOOH-1 at 0.6 V and 0.7 V within 8 h is 52.08% and 69.05%, respectively. The NH_3_ removal rate increases when the applied voltage is higher, which is in agreement with the increased response current for NH_3_ at these voltages. In all the experiments, the concentration of NO_3_^−^ within 8 h is less than 5 mg/L, which can be ignored. However, the accumulation of NO_2_^−^ is observed, as shown in Figure 7b. The conversion rate from NH_3_ to NO_2_^−^ of NiCuOOH-1 at 0.5 V, 0.6 V, and 0.7 V is 16.72%, 17.43%, and 18.22%, respectively. Despite the slight increase in the conversion rate, NiCuOOH-1 exhibits low NO_2_^−^ selectivity of less than 20.00% in the voltage range of 0.5 V to 0.7 V, implying its potential for being utilized in wastewater treatment.

### 2.4. Mechanism Investigation

**Figure 8:**
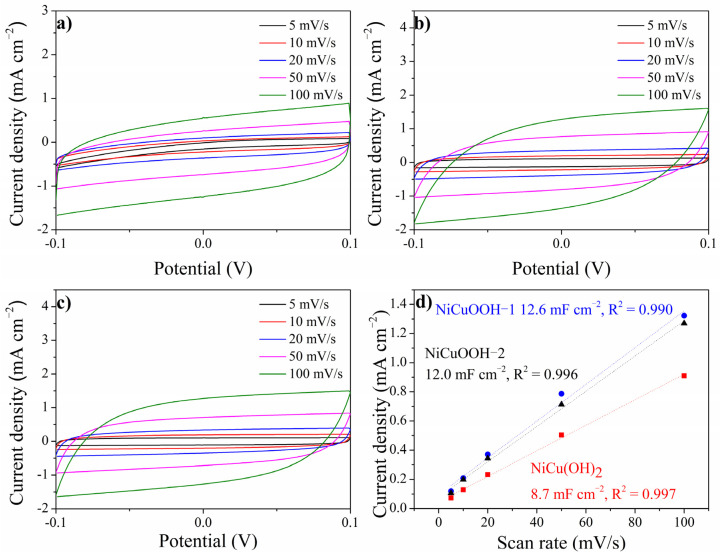
(**a**–**c**) CV measurements at different scan rates (5, 10, 20, 50, and 100 mV/s) of NiCu(OH)_2_, NiCuOOH-1, and NiCuOOH-2, respectively, and (**d**) the calculated Cdl values.

The origin of the good performance of NiCuOOH-1 was further investigated. It is known that the electrochemical surface area (ECSA) plays an important role in the electrochemical reaction, and it has a proportional correlation with the double-layer capacitance (Cdl) [10]. Thus, the Cdl values of NiCu(OH)_2_, NiCuOOH-1, and NiCuOOH-2 were extracted from CV measurements taken at various scan rates in a potential range where no Faradaic reactions occur, as depicted in Figure 8. The Cdl values of NiCu(OH)_2_, NiCuOOH-1, and NiCuOOH-2 were 8.7, 12.0, and 12.6 mF cm^−2^, respectively. The partial pre-transformation from NiCu(OH)_2_ to the NiCuOOH phase significantly improved the Cdl, indicating that NiCuOOH-1 exhibited a larger ECSA than NiCu(OH)_2_. In other words, NiCuOOH-1 has more electrochemical active sites than NiCu(OH)_2_. The NiCuOOH-2 samples, which are treated under a higher oxidation condition, showed similar Cdl compared to NiCuOOH-1. This result suggested that, although the higher oxidation treatment could further improve the crystalline degree, it might not increase the amount of oxyhydroxide sites on the surface. Taking the better performance of NiCuOOH-1 into consideration, these results indicate that the better performance of NiCuOOH-1 could be due to the larger ECSA, which might originate from the partial transformation of NiCu(OH)_2_ into the NiCuOOH phase.

### 2.5. Stability of NiCuOOH-1

**Figure 9:**
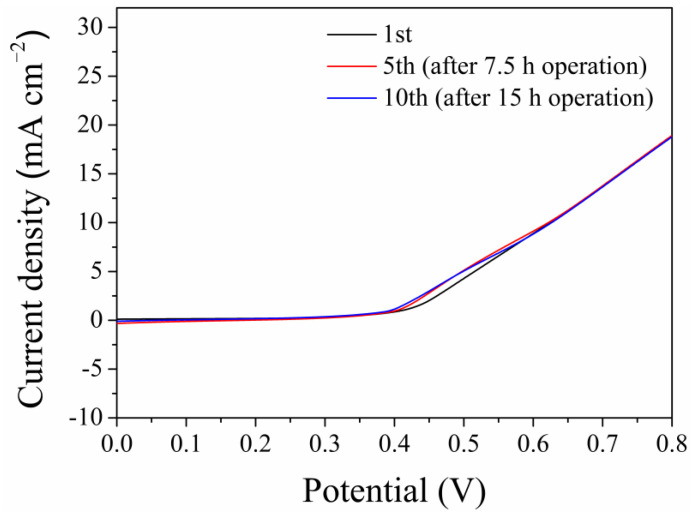
The stability tests of NiCuOOH-1. Each LSV was obtained for the same NiCuOOH-1 sample in fresh 0.1 mol/L KOH + 0.1 mol/L NH_3_.

The stability of electrocatalysts is an important factor for potential practical applications. Hence, the long-term operation by NiCuOOH-1 was conducted. The chronoamperogram of NiCuOOH-1 was performed at 0.5 V for 1.5 h in 0.1 mol/L NH_3_. After that, the solution was replaced to exclude the influence of the change in the ammonia concentration, and the LSV curve was recorded in the fresh solution containing 0.1 mol/L NH_3_. Then, the same NiCuOOH-1 sample was tested for another 1.5 h. As shown in Figure 9, after 10 cycles (15 h of continuous operation), no attenuation could be observed in the recorded LSV curves. The SEM images of the NiCuOOH-1 sample after long-term operation is shown in Figure S5, and the sample retained the similar 3D structure. These results demonstrated the good stability of NiCuOOH-1 in the electrochemical oxidation of ammonia.

## 3. Materials and Methods

### 3.1. Materials

Potassium hydroxide (KOH), potassium persulfate (K_2_S_2_O_8_), nickel sulfate hexahydrate (NiSO_4_·6H_2_O), copper nitrate gerhardite (Cu(NO_3_)_2_·3H_2_O), urea (CO(NH_2_)_2_), and ammonium hydroxide (NH_3_·H_2_O) were purchased from Chongqing Chuandong Co., Ltd. (Chongqing, China). The carbon cloth (type: HCP331N) was obtained from Shanghai Hesen Co., Ltd. (Shanghai, China). All the chemicals were used without further purification.

### 3.2. Synthesis of Working Electrodes with Catalysts

The carbon cloth was cut into pieces (1 × 2 cm^2^) as the substrate electrode. The catalysts were grown onto the carbon cloth during the hydrothermal reaction.

Firstly, one piece of the carbon cloth was put into the reaction kettle, and then, the solution with the volume of 40 mL containing 2 mmol metal ions and 150 mg urea was added. The proportion of Ni and Cu is 8:2 if it is not mentioned. The kettle was sealed and heated at 120 °C for 6 h. After cooling to room temperature, the carbon cloth was cleaned by water and named NiCu(OH)_2_ in this work.

Then, NiCu(OH)_2_ was immersed into 50 mL solution with 1 mol/L KOH and 600 mg K_2_S_2_O_8_. The solution was heated at 60 °C for 2 h to transform NiCu(OH)_2_ into the NiCuOOH phase. The obtained sample was named NiCuOOH-1.

The sample treated by a more powerful oxidizing condition was obtained by a similar method with the solution containing 5 mol/L KOH and 1200 mg K_2_S_2_O_8_. This sample was expected to exhibit a higher crystalline degree of the NiCuOOH phase, and it was named NiCuOOH-2.

### 3.3. Physicochemical Characterization

X-ray diffraction (XRD) patterns were measured on an Ultima IV (Rigaku Corporation, Tokyo, Japan) with the scan rate of 5°/min and the scan range from 10° to 90°. Scanning electron microscopy (SEM) images were obtained on a Hitachi S-4800 (HITACHI, Tokyo, Japan). Transmission electron microscopy (TEM) images were collected on a JEM F200 (JEOL, Tokyo, Japan) with working voltage of 200 kV. X-ray photoelectron spectra (XPS) were obtained with an ESCALAB 250Xi spectrometer using a nonmonochromatized A1 Kα X-ray source (1486.6 eV). The powder of samples was collected from the electrodes for the XRD measurements.

### 3.4. Electrochemical Measurements and Chemical Analysis

Electrochemical measurements were conducted on CHI660E in a three-electrode cell. The saturated Hg/HgO electrode (SCE) was used as a reference electrode, while a Pt sheet was employed as a counter electrode. The electrochemical measurements were conducted without IR-drop correction. All the samples were activated by cyclic voltammetry (CV) test with the scan rate of 100 mV/s in the 1 mol/L KOH solution for more than 20 cycles until the current density is stable. Linear sweep voltammetry (LSV) was performed at the scan rate of 2 mV/s in 0.1 mol/L KOH with or without 0.1 mol/L NH_3_. Electrochemical impedance spectroscopy (EIS) was performed in the frequency range of 0.1–10^5^ Hz in the solution containing 0.1 mol/L KOH and 0.1 mol/L NH_3_. All the electrochemical measurements were performed at room temperature. A two-chamber reactor divided by a Nafion117 membrane was used in the NH_3_ removal experiment to exclude the influence of the counter electrode since the reduction of byproducts on the Pt electrode influences the concentration of both byproducts and NH_3_. The electrode used for the removal of NH_3_ is 5 cm^−2^, and the volume of the solution is 50 mL in one chamber. The ammonium was analyzed by Nessler’s reagent spectrometry, and the detection wavelength was 420 nm. Nitrite was analyzed by a colorimetric assay under the wavelength of 540 nm according to Chinese national standard method GB 7493-87 [41]. Nitrate was also analyzed by a colorimetric assay according to Chinese national standard method GB 11893-89 [42] with the detection wavelengths of 220 nm and 275 nm. For both nitrite ions and nitrate ions, the samples were detected three times in parallel using a UV-2550 ultraviolet spectrophotometer (Shimadzu, Tokyo, Japan).

## 4. Conclusions

In this study, an effective strategy to enhance the activity of NiCu(OH)_2_ for the electrochemical oxidation of ammonia by transforming NiCu(OH)_2_ into the NiCuOOH phase is provided. By treating NiCu(OH)_2_ with an oxidative solution, the partially pre-transformed NiCuOOH electrode presents significantly better performance than NiCu(OH)_2_. Our results indicate that the pre-formation of the NiCuOOH phase can enhance its activity due to the increased number of active sites and the lower charge transfer resistance. However, the sample with a higher crystalline degree of the NiCuOOH phase exhibited lower performance, demonstrating the importance of a moderate treatment condition. Due to the appropriate partial transformation, the optimized NiCuOOH-1 sample presents a high response current density towards ammonia oxidation, low selectivity (<20%) towards NO_2_^−^, and stable activity during the long-term operation. This study demonstrated that the pre-formation of the NiCuOOH phase instead of the in situ generation of amorphous NiCuOOH is beneficial for enhancing the performance, which will aid in the development of transition metal catalysts for the electrochemical oxidation of ammonia.

## Data Availability

Data are contained within the article and Supplementary Materials.

## Supplementary Materials

The following supporting information can be downloaded at: https://www.mdpi.com/article/10.3390/molecules29102339/s1, Figure S1. The XPS survey of NiCu(OH)2, NiCuOOH-1 and NiCuOOH-2, respectively. Figure S2. (a) The high-resolution XPS Cu 2p3/2 spectrum of (a) NiCu(OH)2 and (b) NiCuOOH-2. Figure S3. The LSVs of NiCu(OH)2, with different Ni:Cu ratio in 0.1 mol/L KOH + 0.1mol/L NH3. Figure S4. (a) The LSVs of NiCu(OH)2, NiCuOOH-1, and NiCuOOH-2 in 0.1 mol/L KOH with or without 0.1 mol/L NH3. (b) The Tafel curves calculated from LSVs. Figure S5. (a,b) The SEM images of the NiCuOOH-1 after long-term operation.
