# Supplementary material for: The Enhanced Performance of NiCuOOH/NiCu(OH)2 Electrode Using Pre-Conversion Treatment for the Electrochemical Oxidation of Ammonia
Source: Molecules. 2024 May 16;29(10):2339. doi: 10.3390/molecules29102339 (PMC11124015; doi:10.3390/molecules29102339)
Supplement: Supplementary file 1 [file molecules-29-02339-s001.zip › molecules-2968181-supplementary.pdf]

Supporting Information

The Enhanced Performance of NiCuOOH/NiCu(OH)2 Electrode Using Pre-Conversion Treatment for the Electrochemical Oxidation of Ammonia

Xuejiao Yin [1,*], Jiaxin Wen [1], Jujiao Zhao [2], Ran An [2], Ruolan Zhang [2], Yin Xiong [3], Yanzong Tao [3], Lingxin Wang [4], Yuhang Liu [4], Huanyu Zhou [5] and Yuanyuan Huang [6,7,*]

[1] School of Architecture and Engineering, Chongqing Industry Polytechnic College, Chongqing 401120, China
[2] College of Environment and Resources, Chongqing Technology and Business University, Chongqing 400067, China; zhaojujiao@ctbu.edu.cn (J.Z.)
[3] Chongqing Baihan Wastewater Treatment Co., Ltd., Chongqing 400000, China; 13594739722@163.com (Y.X.)
[4] School of Civil Engineering and Architecture, Chongqing University of Science and Technology, Chongqing 401331, China
[5] Green Intelligence Environmental School, Yangtze Normal University, Chongqing 408100, China
[6] Key Laboratory of Hydraulic and Waterway Engineering of the Ministry of Education, School of River and Ocean Engineering, Chongqing Jiaotong University, Chongqing 400074, China
[7] Chongqing Academy of Science and Technology, Chongqing 401120, China
* Correspondence: yinxuejiao1012@126.com (X.Y.); 18983093831@163.com (Y.H.)

Supplementary figures

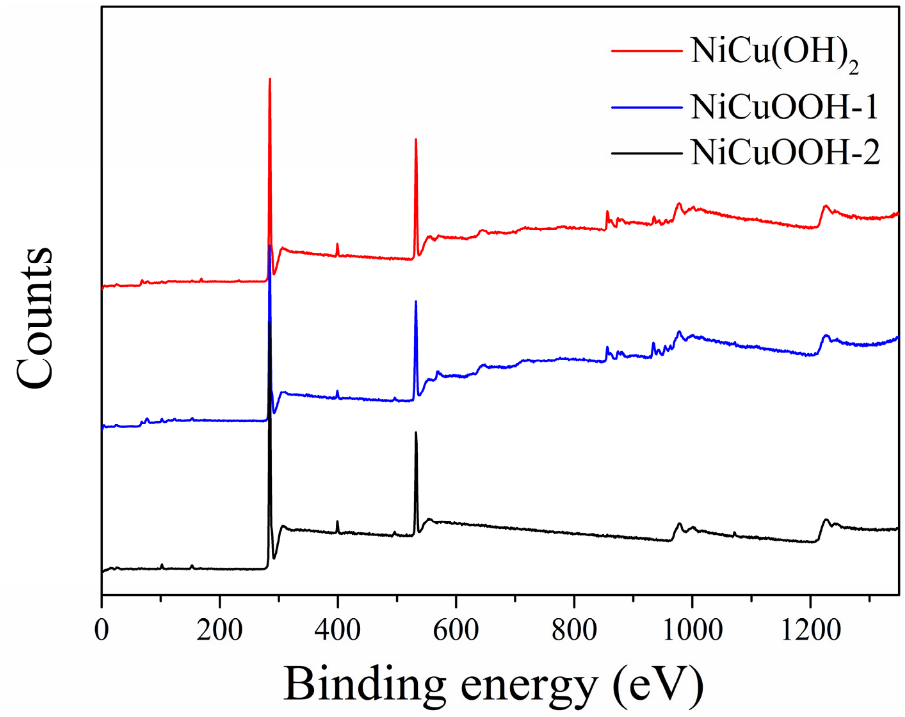

Figure S1. The XPS survey of $NiCu(OH)_2$, NiCuOOH-1 and NiCuOOH-2, respectively.

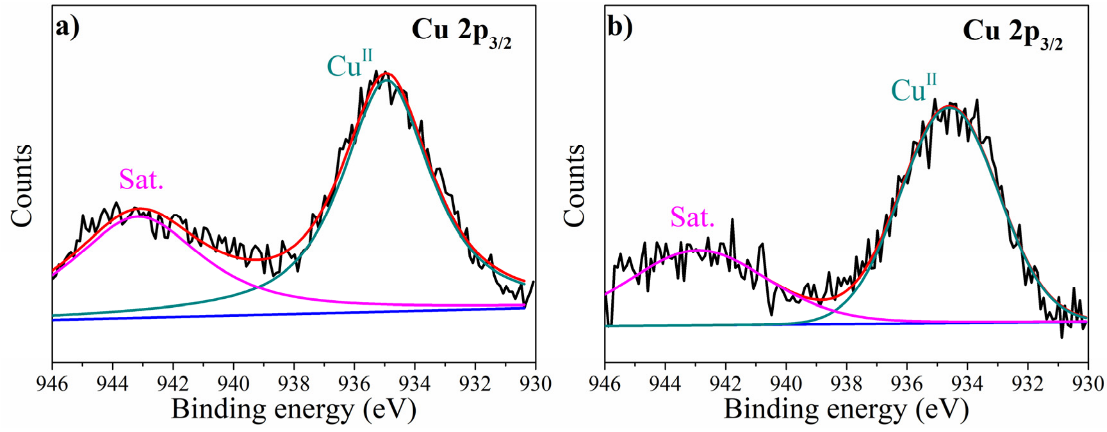

Figure S2. (a) The high-resolution XPS Cu $2p_{3/2}$ spectrum of (a) $NiCu(OH)_2$ and (b) NiCuOOH-2.

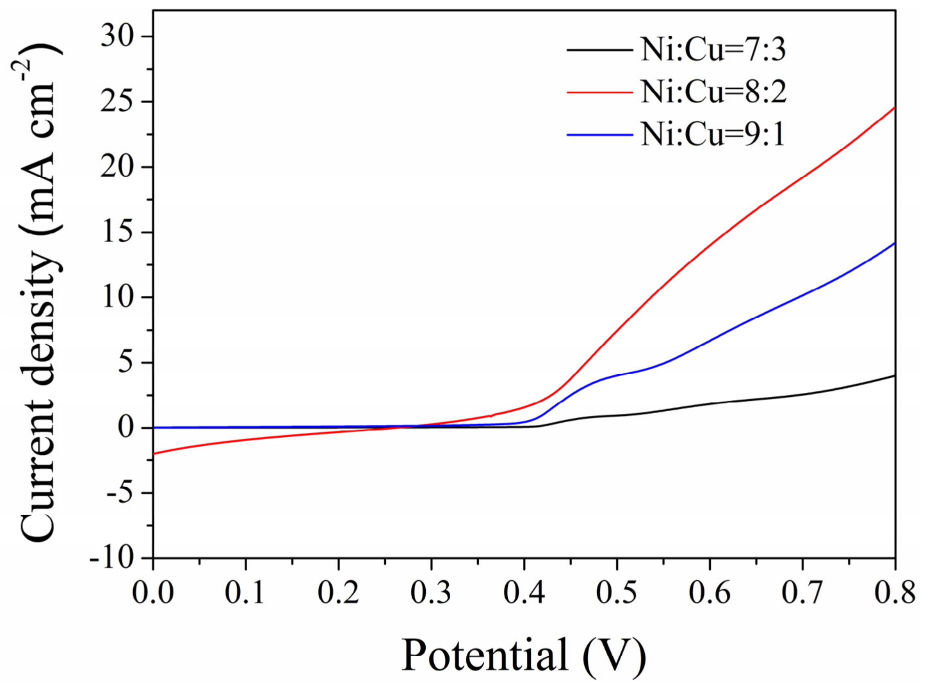

Figure S3. The LSVs of NiCu(OH)$_2$, with different Ni:Cu ratio in 0.1 mol/L KOH + 0.1mol/L NH_3;

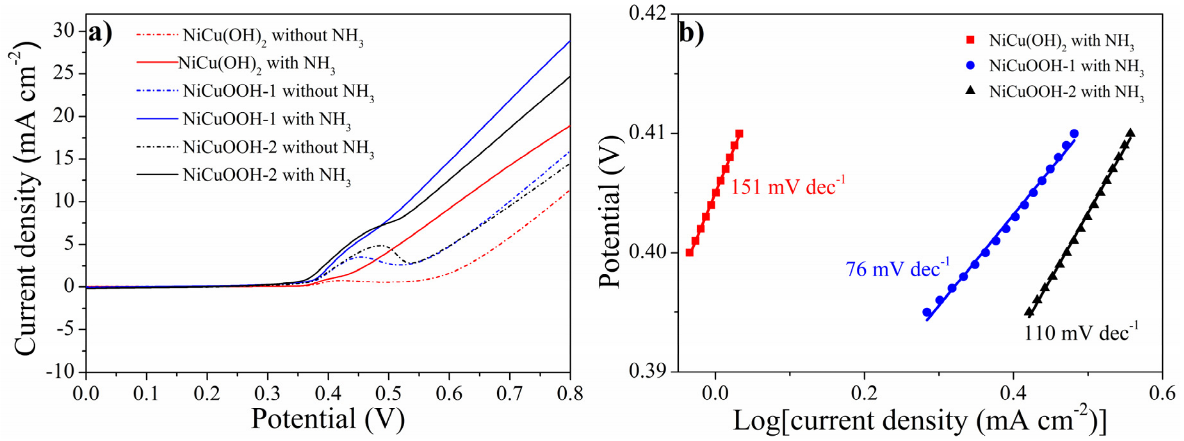

Figure S4. (a) The LSVs of NiCu(OH)$_2$, NiCuOOH-1, and NiCuOOH-2 in 0.1 mol/L KOH with or without 0.1mol/L NH_3.(b) The Tafel curves calculated from LSVs.

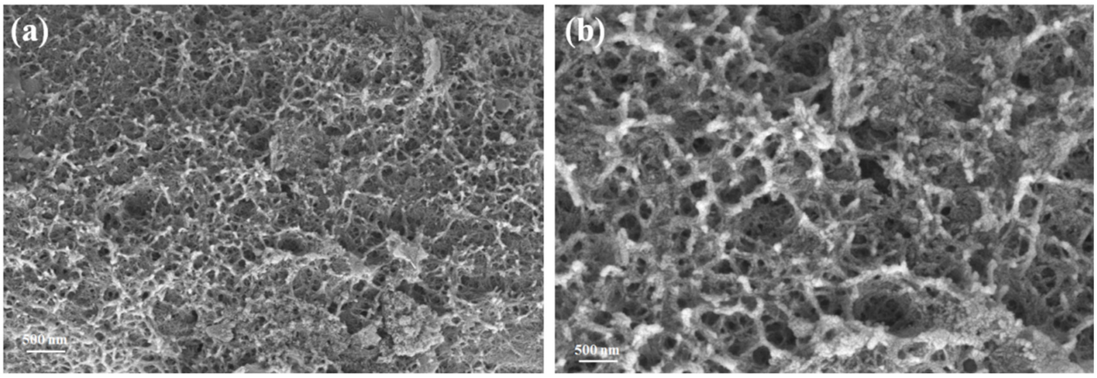

Figure S5. (a, b) The SEM images of the NiCuOOH-1 after long-term operation.,
